# Black cumin (*Nigella sativa*) oleosome as a natural delivery system for curcumin: characterization, preparation, and *in vitro* digestive behavior

**DOI:** 10.3389/fnut.2026.1797551

**Published:** 2026-05-04

**Authors:** Negin Hosseini, Zahra Emam-Djomeh, Gholamreza Askari, Ali Hashemi Nejad, Haniye Takbirgou, Raimar Lobenberg, Neal M. Davies, Michael J. Serpe, Maryam Salami

**Affiliations:** 1 Department of Food Science, Engineering, and Technology, College of Agriculture and Natural Resources, Karaj Campus, University of Tehran, Karaj, Iran; 2 Faculty of Pharmacy and Pharmaceutical Sciences, University of Alberta, Edmonton, AB, Canada; 3 Department of Chemistry, University of Alberta, Edmonton, AB, Canada

**Keywords:** black cumin oleosome, curcumin, pH-shift encapsulation method, *Nigella sativa*, gastrointestinal digestion, bioavailability enhancement

## Abstract

Oleosomes, also known as oil bodies (OBs), are naturally occurring colloidal particles present in edible plant seeds and nuts and possess unique functional properties. In this study, oleosomes derived from black cumin (BCO) were extracted and used as novel, natural carriers for the encapsulation of hydrophobic compounds, using curcumin as a model bioactive. An optimized extraction method achieved a satisfactory recovery yield of approximately 38%, and the physicochemical characteristics of black cumin oleosome (BCO) and its associated proteins were systematically characterized. Curcumin is an unstable bioactive compound of turmeric with high sensitivity to degradation and metabolism, which needs to be encapsulated and protected within a matrix. Curcumin was well encapsulated into BCO using a different pH-shift method, resulting in a high encapsulation efficiency of 91%, without precipitation of curcumin, which was consistent with the FT-IR results, indicating that 10 mg of curcumin was successfully encapsulated within the interior core of oleosomes. Curcumin-loaded oleosomes demonstrated improved functional performance, including enhanced antioxidant stability and emulsifying properties. *In vitro* gastrointestinal digestion studies revealed a cumulative curcumin release of 64% by the end of the intestinal phase, confirming the effectiveness of the pH-shift encapsulation method and the black cumin oleosome as a vehicle for delivering curcumin and increasing its bioavailability. Overall, these findings indicate that curcumin could be well encapsulated within naturally derived oleosomes via a novel pH-shift method and black cumin oleosomes are effective carriers for enhancing the delivery of hydrophobic nutraceuticals via oral routes, which shows strong potential for incorporation of oleosomes into existing food formulations or for the development of novel functional food products.

## 1 Introduction

**Figure 1:**
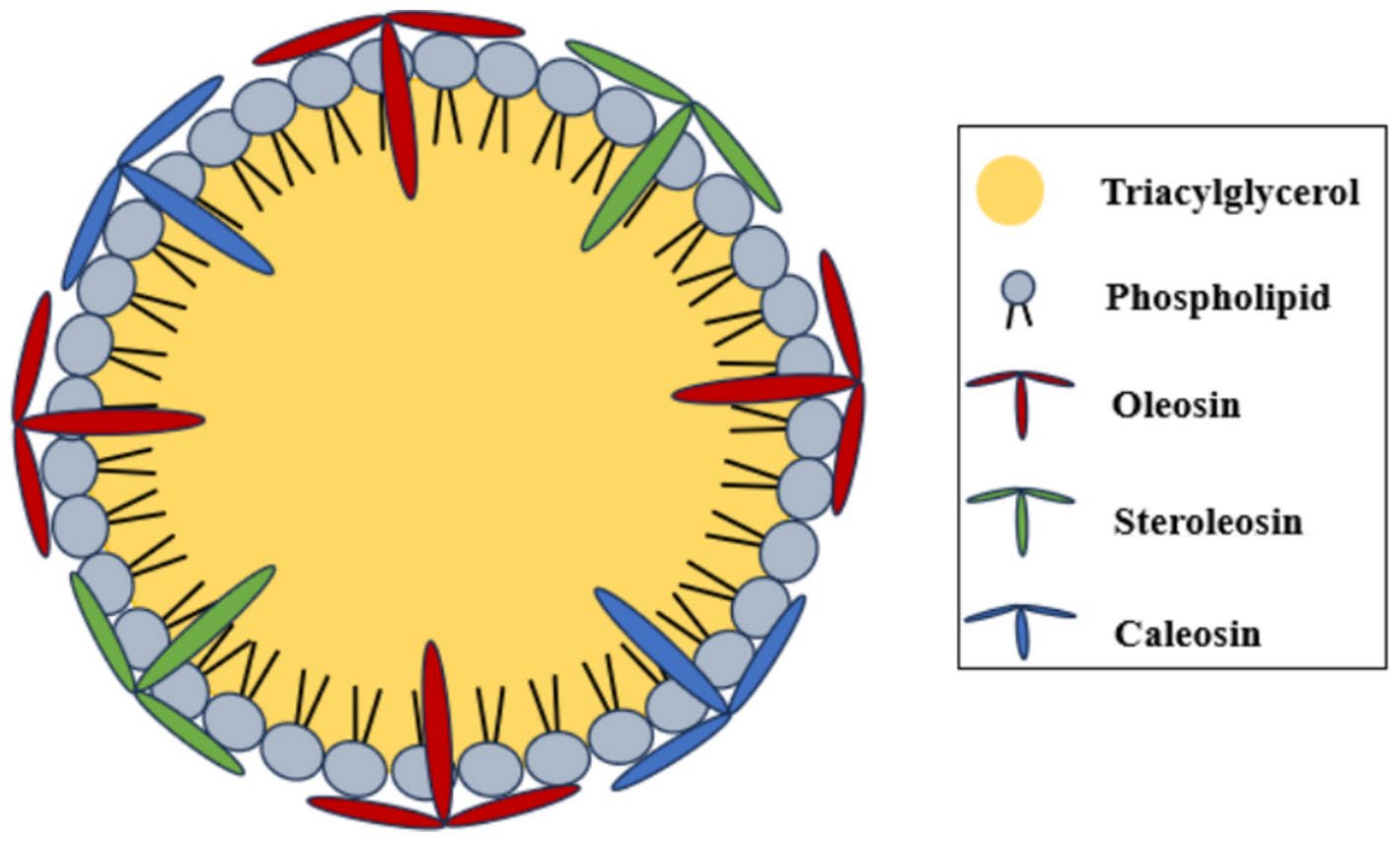
Proposed oleosome structure.

Plant-based ingredients have recently attracted increasing interest due to their well-documented health-promoting properties and environmental benefits. In response, the food industry is actively reformulating existing products by replacing animal-derived and synthetic ingredients with plant-based alternatives. Oleosomes, also referred to as oil bodies or oil droplets, are natural lipid storage organelles commonly found in plant seeds, particularly in oil-rich (oleaginous) species (1–3). These structures are micron-sized colloidal particles, typically ranging from 0.3 to 4 μm in diameter (most often 0.5–2.5 μm), and consist of a hydrophobic core of triacylglycerols (TAGs) surrounded by a thin hydrophilic membrane composed of a phospholipid monolayer embedded with unique structural proteins, namely oleosin, caleosin, and steroleosin (4, 5) (Figure 1).

Membrane-associated proteins act as molecular anchors that maintain oleosome integrity by balancing membrane flexibility and rigidity (6). In addition, densely packed phospholipids and proteins form an effective barrier that protects the internal TAGs from environmental stresses, particularly oxidative degradation, eliminating the need for added antioxidants (7, 8). The stability of oleosomes against aggregation is governed by their surface chemistry and the surrounding environmental conditions (9, 10). Their intrinsic negative surface charge and micron-scale size prevent coalescence, making oleosomes stable, discrete droplets suitable for delivery of labile nutraceuticals within food matrices, thereby enhancing bioavailability (11). The hydrophobic core of oleosomes provides an ideal environment for solubilizing and transporting non-polar bioactive compounds, including oil-soluble vitamins and nutraceuticals. Moreover, the presence of phospholipids and proteins enables oleosomes to spontaneously form oil-in-water emulsions without the use of emulsifiers or high-pressure homogenization. Owing to these distinctive characteristics and market need for natural carriers, oleosomes have gained considerable attention for a wide range of food and nutraceutical applications.

Curcumin, the principal bioactive compound in turmeric, exhibits a wide range of biological activities, including anti-inflammatory, antimicrobial, and anticarcinogenic effects (1, 12, 13). However, its practical application is limited by poor water solubility, low bioavailability, and high susceptibility to degradation and metabolism. To address these limitations, various encapsulation strategies have been developed, particularly those involving oil-in-water emulsions to enhance curcumin and its bioactive compounds’ stability, controlled release of curcumin from carriers, and bioavailability (14). One commonly used approach involves incorporating curcumin into emulsions followed by thermal treatment to promote its migration into the hydrophobic core (15, 16). However, elevated processing temperatures may induce droplet aggregation and compromise emulsion stability. Alternatively, curcumin can be dissolved in organic solvents, such as ethanol, before encapsulation into pre-formed colloidal systems; nevertheless, this approach incurs additional costs for solvent removal and raises environmental concerns (17, 18).

Recently, a simple, solvent-free pH-shift method has been developed for encapsulating curcumin into various colloidal delivery systems. This approach exploits the pH-dependent solubility of curcumin and pH-induced conformational changes in membrane-associated proteins. At acidic to neutral pH values (pH < 8), curcumin exists predominantly in a neutral, non-polar form with limited water solubility. At higher pH values, curcumin becomes ionized, increasing its polarity and aqueous solubility. In the mentioned method, the encapsulation process typically involves three steps: (1) dissolving curcumin in a highly alkaline solution, (2) mixing this solution with an acidified colloidal suspension, and (3) adjusting the final pH to neutral or below. In the present study, a new, modified pH-shift approach was applied. Specifically, the pH of the oleosome emulsion was first increased to approximately 12, followed by the addition of curcumin powder, after which the pH of the mixture was rapidly reduced to near-neutral values.

Previous studies have successfully encapsulated curcumin, a lipophilic nutraceutical, into oleosomes derived from various plant sources, achieving high encapsulation efficiencies. However, to date, no studies have reported the loading of curcumin into oleosomes derived from black cumin. Black cumin, also known as black seed, is obtained from the medicinal plant *Nigella sativa* L. and is widely recognized for its remarkable therapeutic properties, including gastroprotective, antioxidant, anti-inflammatory, antihypertensive, immunomodulatory, antimicrobial, nephroprotective, neuroprotective, antidiabetic, anticancer, cardioprotective, and hepatoprotective effects (19). These health benefits are largely attributed to the presence of thymoquinone (TQ) and other bioactive phytochemicals, particularly in black cumin oil (20). Rich in protein and fat, and containing appreciable levels of essential fatty acids, minerals, amino acids, and vitamins, black cumin is considered a high-value functional food ingredient (21). Based on these attributes and not being used as a carrier previously, this study aimed to exploit black cumin-derived oleosomes as natural carriers for curcumin delivery. Oleosomes were extracted intact with minimal structural disruption using a modified version of the method described by Vardar et al. (22), and were used as a novel source of carrier for curcumin to protect and increase its bioavailability into food matrices.

The objectives of this study were to characterize native and curcumin-loaded black cumin oleosomes and to evaluate the applicability of the modified extraction and encapsulation methods. Furthermore, the gastrointestinal fate of encapsulated curcumin was investigated using a standardized *in vitro* digestion model. The findings of this work are expected to support the development and reformulation of functional food products enriched with black cumin-derived oleosomes as natural ingredients and efficacious nutraceuticals.

## 2 Materials and methods

### 2.1 Materials

Black cumin (*Nigella sativa* L.) seeds were obtained from local markets in Karaj and Tehran, Iran. Curcumin powder was purchased from BIO BASIC Inc. (Markham, Ontario, Canada). Sodium hydroxide (NaOH), hydrochloric acid (HCl), sodium bicarbonate (NaHCO₃), and all other reagents used in this study were of analytical grade and purchased from Merck.

### 2.2 Methods

#### 2.2.1 Modified extraction of black cumin oleosomes

Black cumin oleosomes were extracted using an alkaline extraction method based on the protocol described by Vardar et al. (22), with minor modifications. An alkaline sodium bicarbonate–carbonate buffer (pH 9.2) was prepared by dissolving 1 mol L^−1^ sodium bicarbonate (NaHCO₃) and adjusting the pH with sodium hydroxide (NaOH).

A known quantity of black cumin seeds was thoroughly washed several times with distilled water and subsequently soaked overnight in the prepared alkaline buffer at a solid-to-liquid ratio of 1:5 (w/v) under continuous stirring (23). After soaking, the seeds were combined with a fresh portion of bicarbonate buffer at a ratio of 1:2 (w/v) and homogenized using a high-speed blender for 5 min in an intermittent mode (30 s on/30 s off). To minimize potential thermal damage caused by frictional heating during blending, an additional 50 mL of buffer was gradually added during the homogenization process.

The resulting slurry was filtered through a filter cloth to remove insoluble residues. Subsequently, three additional volumes of the alkaline buffer were poured through the filter to maximize the recovery of the oleosome-rich cream. The filtrate was then centrifuged at 10,000 × *g* for 30 min at 4 °C to separate the upper oleosome-rich cream layer. To further purify the oleosomes, the collected cream was resuspended in bicarbonate buffer at a ratio of 1:4 (w/v) and centrifuged again under the same conditions. The final oleosome cream fraction was carefully collected and stored at 4 °C for subsequent analyses and encapsulation experiments.

### 2.3 Composition analysis

The oil and protein contents of black cumin seeds were determined using the Soxhlet extraction method (using Tecator, SOXTEC SYSTEM HT 1043 Extraction Unit, Sweden) and the Kjeldahl method (using Tecator, KJELTEC AUTO 1030 Analyzer, Sweden), respectively. The moisture content of the extracted oleosomes was determined by oven-drying at 60 °C for 48 h and was expressed as the percentage of water removed per gram of wet oleosome cream.

For oleosome composition analysis, oil content was measured by Soxhlet extraction using hexane as the solvent, while protein content was determined using the Kjeldahl method. The oil content of oleosomes was reported on a wet weight basis of the oleosome cream. The oil content was calculated using Equation 1:


$$\text{Oil content}\;(\mathrm{wt}\%)=100\times \frac{\text{extracted oil}\;(g)}{\text{initial sample}\;(g)}$$ (1)

### 2.4 Preparation of curcumin-encapsulated oleosomes

Curcumin-loaded oleosomes were prepared using a modified pH-shift method as previously described (24). Briefly, oleosome emulsions (12.5%, w/w) were prepared by dispersing oleosome cream in distilled water and stirring for 60–90 min until a homogeneous suspension was obtained. The pH of the emulsion was gradually increased from approximately 9–12 by dropwise addition of sodium hydroxide (NaOH) over 20–30 min. Curcumin powder was then added at different concentrations (0.002, 0.004, 0.010, 0.020, and 0.040 g) to produce emulsions with varying curcumin loadings. To minimize curcumin degradation under alkaline conditions, the mixtures were stirred gently for 5–7 min.

Following curcumin addition, a predetermined volume of distilled water was added, and the pH of the mixture was reduced to neutral (approximately pH 7) by dropwise addition of hydrochloric acid (HCl) over 15–20 min. The emulsions were subsequently stirred at room temperature for an additional 15 min and then refrigerated for 15 min to facilitate phase stabilization. Finally, curcumin-loaded oleosome cream was isolated by centrifugation at 10,000 × *g* for 30 min at 4 °C and stored at 4 °C for further analyses.

### 2.5 Encapsulation properties

#### 2.5.1 Determination of curcumin concentration

The concentration of curcumin encapsulated in black cumin oleosomes was determined using UV–visible spectrophotometry (SP-UV 500DB, Spectrum Instruments). A standard calibration curve of curcumin was prepared at a wavelength of 426 nm. To extract encapsulated curcumin, curcumin-loaded oleosome creams were mixed with ethanol and subjected to ultrasonication for 30 min. The resulting mixtures were then centrifuged at 9,000 × *g* for 10 min at 4 °C to separate the supernatant containing dissolved curcumin. Curcumin concentration was quantified using the standard calibration curve (25).

#### 2.5.2 Encapsulation efficiency (EE)

The encapsulation efficiency (EE) of curcumin was calculated using Equation 2:


$$\mathrm{EE}\%=100\times C_{\text{encapsulated}}/C_{\text{initial}}$$ (2)

Here, *C*_initial_ represents the total concentration of curcumin initially added to the oleosome emulsion, and *C*_encapsulated_ denotes the concentration of curcumin successfully loaded into the black cumin oleosomes following the pH-shift treatment.

#### 2.5.3 Protein characterization by SDS–PAGE

##### 2.5.3.1 Protein extraction

Oleosome cream was mixed with acetone at a ratio of 1:3 (g/mL) and vortexed for 30 min at room temperature to ensure thorough dispersion. The mixture was then centrifuged at 11,000 × *g* for 10 min at 4 °C to collect the protein-containing precipitate. This extraction step was repeated twice to ensure complete removal of residual esters. The final precipitate was dried under vacuum at room temperature to eliminate residual acetone, freeze-dried, and stored for subsequent analyses (26).

##### 2.5.3.2 SDS–PAGE analysis

The protein profiles of black cumin oleosomes following extraction and washing with bicarbonate buffer, and after simulated gastrointestinal digestion were analyzed using sodium dodecyl sulfate–polyacrylamide gel electrophoresis (SDS–PAGE) according to the method described by Wang et al. (27), with minor modifications. Briefly, freeze-dried protein samples were dissolved in phosphate buffer (pH 8) at various concentrations. Aliquots (100 μL) of each sample were mixed with 50 μL of sample loading buffer containing glycerol, Tris–HCl (pH 6.8), SDS, β-mercaptoethanol, bromophenol blue, and EDTA, and vortexed for 5 min. The mixtures were then heated at 100 °C for 5 min to ensure protein denaturation.

A volume of 40 μL of each prepared sample and 8 μL of a pre-stained protein marker were loaded onto the gel. The stacking and separating gels contained 4 and 15% (w/v) polyacrylamide, respectively. Following electrophoresis, protein bands were stained with Coomassie Brilliant Blue R-250 for 24 h under gentle agitation. The gel was destained six times with destaining solution and imaged using a Samsung S23 Ultra smartphone.

### 2.6 Particle characterization

#### 2.6.1 Particle size distribution

Particle size distribution (PSD) and mean surface-weighted diameter (*D*_32_) were measured in triplicate using a Malvern Mastersizer 2000 (Malvern Instruments Ltd., UK) at 25 °C. Results are reported as the mean of three measurements. Prior to analysis, samples were diluted 25–250-fold with double-distilled water adjusted to the same pH as the sample aqueous phase (pH 7 or 9). The analyzed samples included native-extracted oleosomes (pH 9) and curcumin-loaded oleosomes (pH 7) (12).

#### 2.6.2 *ζ*-potential measurements

The electrical surface properties of the particles were evaluated by measuring electrophoretic mobility using a Zetasizer instrument (Malvern Instruments, UK). Samples were diluted 500–650-fold with double-distilled water at the same pH used for PSD measurements prior to analysis. All measurements were conducted at 25 °C (28).

#### 2.6.3 Confocal laser scanning microscopy (CLSM)

Confocal laser scanning microscopy (CLSM) was employed to visualize the microstructure of curcumin-loaded oleosomes. A 20 μL aliquot of the diluted sample was placed on a microscope slide and covered with a coverslip prior to observation.

#### 2.6.4 Fourier transform infrared (FT–IR) spectroscopy

FT–IR spectra were recorded using an FT–IR spectrometer (FT-IR Spectroscopy, VECTOR33, Bruker Co., Germany). Samples were prepared using the KBr pellet method, and 45 scans were collected over a spectral range of 4,000–400 cm^−1^ at a resolution of 4 cm^−1^ (1).

#### 2.6.5 Intrinsic fluorescence emission spectroscopy

Intrinsic fluorescence spectra were recorded with slight modifications to previously reported methods. Unloaded and curcumin-loaded oleosome creams were diluted with ultrapure water to a final concentration of 0.02 mg mL^−1^. Fluorescence measurements were performed using a fluorescence spectrophotometer (Agilent Technologies, USA). Emission spectra were recorded in the ranges of 300–450 nm and 400–600 nm with excitation wavelengths of 280 nm and 420 nm, corresponding to oleosome proteins and curcumin, respectively. The excitation and emission slit widths were set to 5 nm and 10 nm, respectively (29).

### 2.7 Antioxidant capacity assay

The antioxidant capacity of extracted, purified, and curcumin-loaded oleosome creams was evaluated using the 2,2′-azino-bis(3-ethylbenzothiazoline-6-sulfonic acid) (ABTS) radical scavenging assay with minor modifications (30). The ABTS^+^ radical solution was prepared by mixing ABTS with potassium persulfate (K₂S₂O₈) and stored in the dark at 4 °C for 24 h prior to use. For sample preparation, oleosome creams were diluted with 80% methanol at a ratio of 1:10 (g/mL) and stirred for 2 h at room temperature. The ABTS stock solution was diluted with phosphate buffer (5 mM, pH 7.4) to obtain an absorbance of 0.70 ± 0.02 at 734 nm. The diluted samples were then mixed with the adjusted ABTS solution at a fixed ratio and incubated in the dark for 7 min at room temperature. Absorbance was measured at 734 nm using a UV–visible spectrophotometer (SP-UV 500DB, Spectrum Instruments), and radical scavenging activity was calculated using Equation 3:


$$\text{ABTS free radical scavenging ability}\;(\%)=\frac{A_{1-}A_{2}}{A_{1}}\times 100$$ (3)

Where *A*_1_ and *A*_2_ are the absorbance of the blank and sample, respectively.

### 2.8 Emulsifying properties

The emulsion activity index (EAI) and emulsion stability index (ESI) of the samples were determined following the method of Li et al. (31), with minor modifications. Briefly, oleosome samples were diluted with water at a ratio of 1:10 (g/mL) and stirred continuously for 90–120 min to ensure complete dispersion. Oil was then incorporated at a 1:3 (v/v) ratio, and the mixture was homogenized for 2 min using a high-speed homogenizer (Metabo, Germany).

Aliquots (50 μL) were collected from the bottom of the emulsion immediately after homogenization (0 min) and after 20 min. Each aliquot was subsequently diluted 100-fold with a 10% (w/v) sodium dodecyl sulfate (SDS) solution. The absorbance of the diluted samples was measured at 500 nm using a UV–visible spectrophotometer (SP-UV 500DB, Spectrum Instruments).

The EAI and ESI were calculated using Equations 4, 5:


$$\mathrm{EAI}\;(m^{2}/g)=2\times 2.303\times A_{0}\times N/C\times \varphi \times 10^{4}$$ (4)

$$\mathrm{ESI}\;(\min)=(A_{0}/\mathrm{\Delta A})\times t$$ (5)

In these equations, *A*_0_ represents the absorbance at time 0, Δ*A* is the change in absorbance over 20 min, *N* is the dilution factor, *C* is the protein concentration per unit volume (g/mL), *φ* is the volume of oil used in the emulsion, and *t* corresponds to the 20-min time interval.

### 2.9 Simulated gastrointestinal tract (GIT) digestion

The standardized INFOGEST method was used to simulate the human gastrointestinal environment and investigate changes in oleosome protein composition as well as the fate of curcumin (32). All simulated digestive fluids were preheated to 37 °C and mixed with the sample at specified ratios. Mixtures were incubated at 37 °C with gentle shaking at 100 rpm for the designated times.

#### 2.9.1 Oral phase

Curcumin-loaded oleosome cream was mixed with simulated saliva fluid (SSF) containing CaCl₂ at a 1:1 (w/w) ratio. The mixture was incubated for 2 min to simulate oral digestion.

#### 2.9.2 Gastric phase

The oral mixture was combined with simulated gastric fluid (SGF) containing CaCl₂ at a 1:1 (v/v) ratio. The pH was adjusted to 3, and pepsin was added to a final activity of 2000 U/mL. The mixture was incubated for 2 h at 37 °C.

#### 2.9.3 Small intestinal phase

Gastric digesta was mixed with simulated intestinal fluid (SIF) at a 1:1 (v/v) ratio, containing CaCl₂. Bile salts and enzyme solutions (pancreatin and lipase) were added to achieve final concentrations of 10 mM, 100 U/mL, and 2000 U/mL, respectively. The pH was adjusted to 7, and the pH-stat method was applied to maintain this value throughout incubation. The intestinal phase lasted 90 min.

#### 2.9.4 Determination of curcumin release rate

The release of curcumin during gastrointestinal digestion was monitored using the method described by Zhu et al. (33). Aliquots of digesta were collected every 30 min and centrifuged at 12,000 × *g* for 15 min. The cumulative release of curcumin was calculated using Equation 6:


$$\text{Curcumin release}\;(\%)=\frac{C_{\text{supernatant}}}{C_{\text{initial}}}\times 100\;$$ (6)

In this equation, *C*_supernatant_ represents the curcumin concentration in the supernatant, and *C*_initial_ denotes the initial curcumin content in the undigested sample.

### 2.10 Statistical analysis

All experiments were performed in triplicate or more. Data are presented as mean ± standard deviation (SD) with a 95% confidence level (*p* < 0.05). Plots were prepared using Adobe Photoshop 2022 (v23.5.4.981). Statistical analyses were performed using the Microsoft Excel and SPSS software package (SPSS Inc., Chicago, IL, USA).

## 3 Results and discussion

### 3.1 Compositional characterization of oleosomes

Black cumin seeds contained 42.8 wt% oil. Using the modified extraction procedure, the maximum yield of oleosome cream was 38.53 wt%. The oil content of the extracted oleosomes was 20.98% (w.b.), while the seeds contained around 20.65% protein, of which 9.193% was recovered in the oleosome fraction. The moisture content of the oleosomes was approximately 46 wt%. Oleosin, the most abundant protein in oleosomes, has an isoelectric point around pH 5 (14). Extraction under alkaline conditions (pH 9.2) deprotonates the carboxylic groups of oleosome proteins, resulting in a negative *ζ*-potential. Compared to rapeseed-derived oleosomes (protein content ~3 wt%, *ζ*-potential −37 mV), black cumin oleosomes exhibited a higher negative charge of −54 mV. This is likely due to the higher protein content of black cumin oleosomes, as previously reported by Zhang et al. (34).

### 3.2 Encapsulation efficiency of curcumin in oleosomes

**Figure 2:**
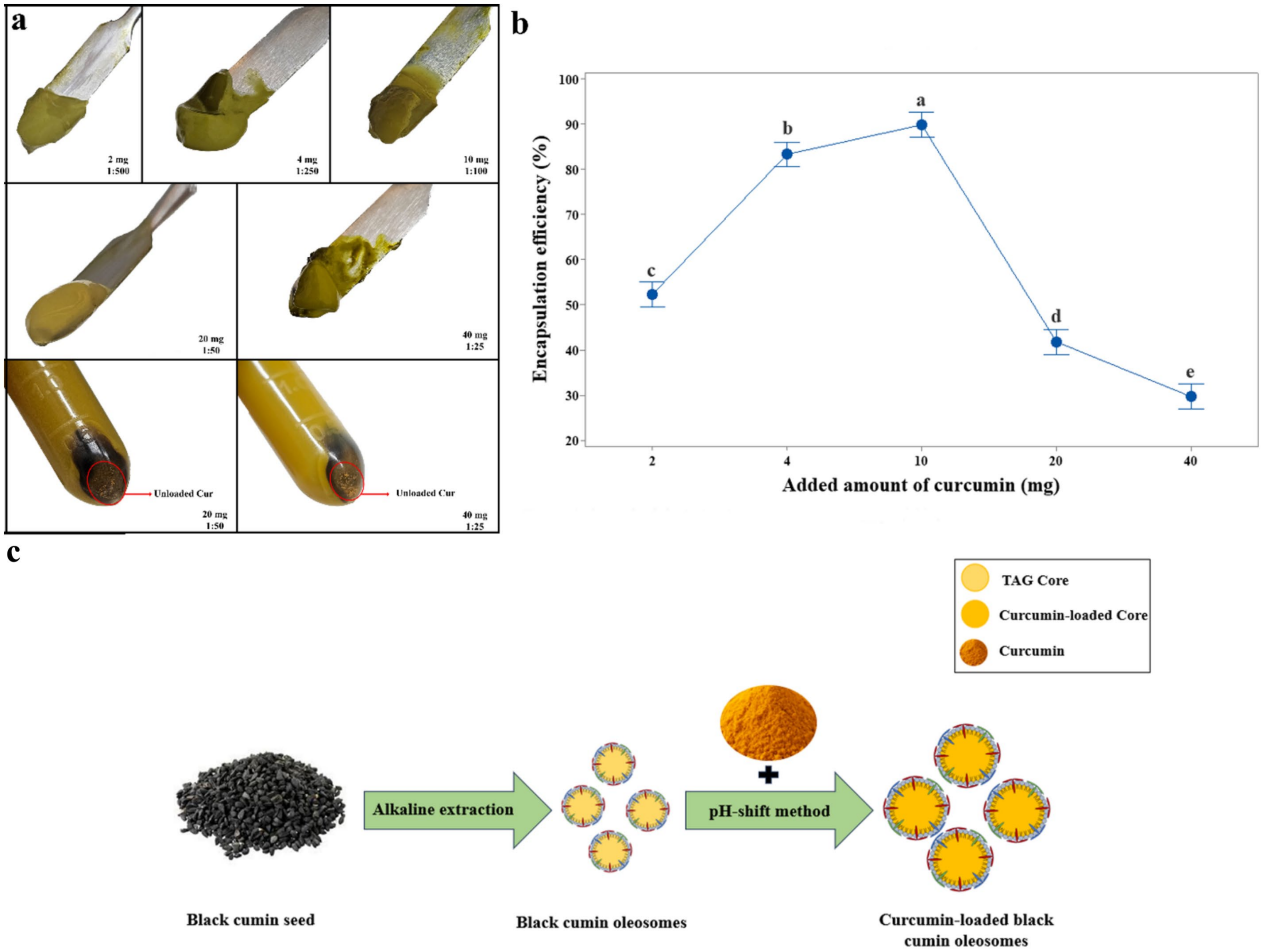
**(a)** Appearance of curcumin-loaded oleosome creams. **(b)** The encapsulation efficiency of curcumin in oleosomes at different additive amounts of curcumin (cur) (*p* < 0.05). **(c)** Schematic mechanism for the formation of curcumin-loaded black cumin oleosomes.

Encapsulation efficiency is an important factor that shows the effectiveness of the construction of a bioactive compound delivery system. Curcumin was encapsulated using a pH-shift treatment, leveraging changes in curcumin’s hydrophobicity and water solubility, along with structural changes in oleosome membrane proteins, consistent with intrinsic fluorescence result. At pH ~12, proteins unfold; upon lowering the pH to 7, curcumin transitions from water-soluble to oil-soluble, migrating into the oleosome core. In this study, various curcumin amounts were tested to determine the highest amount of curcumin. As the curcumin content increased, the turbidity in the lower solution was observed after centrifugation. Moreover, the precipitation of free curcumin occurred when the curcumin content reached 20 mg and 40 mg (Figure 2a). Encapsulation efficiencies (EE%) decreased when the curcumin content increased. This was likely due to the saturation of the encapsulation core of oleosome and overflowing beyond the core’s loading threshold; when the curcumin exceeds the oleosome’s entrapment capacity, unencapsulated curcumin is likely lost outside the oleosome. These observations were consistent with the reports by Wei et al. (2), Ding et al. (3), and Nikiforidis and Kiosseoglou (4). Encapsulation efficiency percentages were (52.22 ± 2.66), (83.21 ± 2.31), (89.75 ± 1.059), (41.75 ± 2.81), and (29.619 ± 1.242) for 2, 4, 10, 20, and 40 mg curcumin, respectively (Figure 2b). The highest EE ( ~ 90%) without any precipitation occurred at 10 mg curcumin (*W*_curcumin_: *W*_oleosome_ = 1%), which suggests the black cumin oleosome and pH-shift method being an effective system and method for delivering curcumin being poor water soluble. Thus, the optimum amount of curcumin was selected as 10 mg for the subsequent experiments.

### 3.3 Physicochemical characterization of native and curcumin-loaded oleosomes

#### 3.3.1 Protein profile

**Figure 3:**
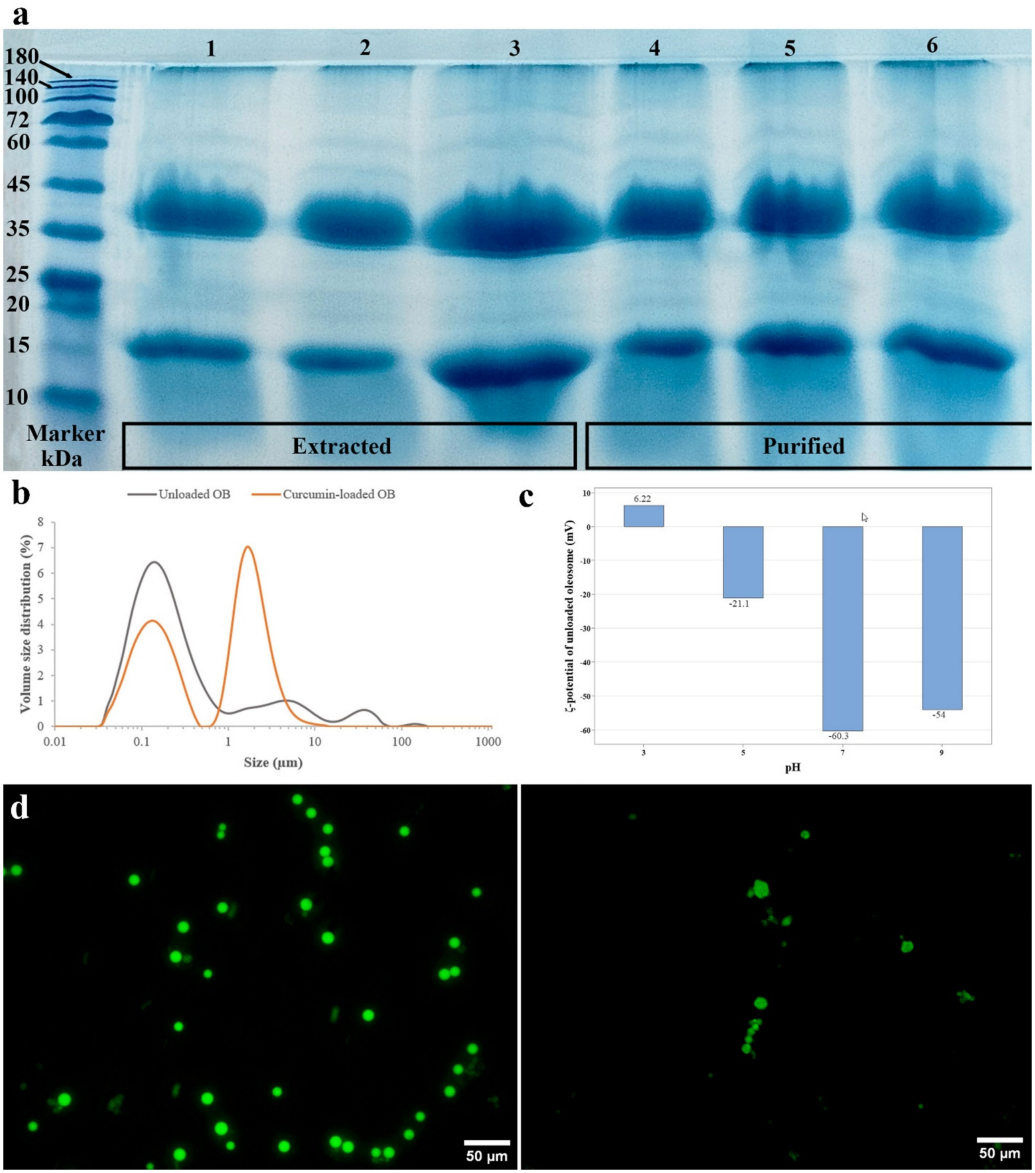
Structural characterization of unloaded- and curcumin-loaded oleosomes. **(a)** SDS-PAGE image of the oleosome (OB) membrane protein: Marker; proteins from extracted oleosomes; proteins from purified oleosomes (1 and 4, 2 and 5, 3 and 6 correspond to 20,000, 25,000, 30,000 ppm, respectively). **(b)** The particle distribution spectrum. **(c)** Zeta potential. **(d)** Confocal laser scanning microscopy of curcumin-loaded oleosomes at pH 7.

SDS–PAGE analysis revealed the most abundant membrane proteins at 15–26 kDa (oleosins) (9), with higher molecular weight proteins corresponding to steroleosins (>35 kDa) and caleosins (27–35 kDa) (35). Figure 3a shows that protein composition remained unchanged after washing and purification, confirming the structural integrity of black cumin oleosome, consistent with the reports by Ntone et al. (36).

#### 3.3.2 Particle size distribution

The mean particle size of native black cumin oleosomes (BCOs) was 0.165 μm, displaying a unimodal distribution. Curcumin loading increased the mean size to 0.244 μm and produced a bimodal distribution (~0.2 μm and ~2.5 μm), consistent with confocal laser scanning microscopy (CLSM) observations, which displays some droplets being aggregated (Figures 3b,d). Similarly, Cuomo et al. (37) and Wei et al. (2) found that curcumin addition to oleosomes results in an increase in mean particle size of native oleosomes. This is because of the addition of curcumin to the oil phase of the oleosomes. Another reason may be due to the destabilizing effects of the pH shifts on the protein-phospholipid membrane of oleosomes. At pH values near the isoelectric point (pI) of proteins, there are lower repulsive forces between the oleosome droplets, followed by association of droplets, causing the droplets become bigger; when the pH decreased to 7, the *ζ*-potential decreased from −54 mV to −35 mV, confirming the high sensitivity of oleosomes to pH and having bigger sizes observed by confocal microscopy (Figure 3d).

#### 3.3.3 *ζ*-potential

Native black cumin oleosome had a *ζ*-potential of −54 mV at pH 9, which decreased to −35 mV after curcumin loading at pH 7 (Figure 3c). According to Bhatla et al. (5) and Qi et al. (9), under alkaline pH values, the carboxyl and amino groups of the oleosome proteins are deprotonated, increasing the repulsive forces between charges and therefore resulting in the unfolding of surface protein structures. Thus, some of the ionized groups of proteins become exposed, which results in a higher *ζ*-potential, and when the pH is returned to neutral, the repulsive forces become weakened. As a result, the surface proteins fold again, and the ionized groups are less exposed, which leads to a decrease in *ζ*-potential. This may lead to conformational changes in membrane proteins and partial coalescence, which was consistent with the intrinsic fluorescent results and CLSM observations, respectively (38, 39).

#### 3.3.4 FT-IR analysis

Black cumin oleosomes displayed characteristic peaks at 2927, 2853 cm^−1^ (C–H stretching), and 1,655, 1,424, 721 cm^−1^ (C=O stretching, C–H deformation, CH₂ groups) (40). Free curcumin showed peaks at 3509, 1505, 1,294, and 1,025 cm^−1^, corresponding to O–H, C–C, C=CH bending, and C–O–C stretching, respectively (41). However, after encapsulation, characteristic curcumin peaks diminished or slightly shifted. As Mohammadian et al. (42) have reported, this phenomenon may be due to the carrier (oleosome, in this case) concealing these typical peaks by limiting the stretching and bending of curcumin bonds, which indicates successful entrapment without altering intrinsic properties (43).

#### 3.3.5 Intrinsic fluorescence spectra

**Figure 4:**
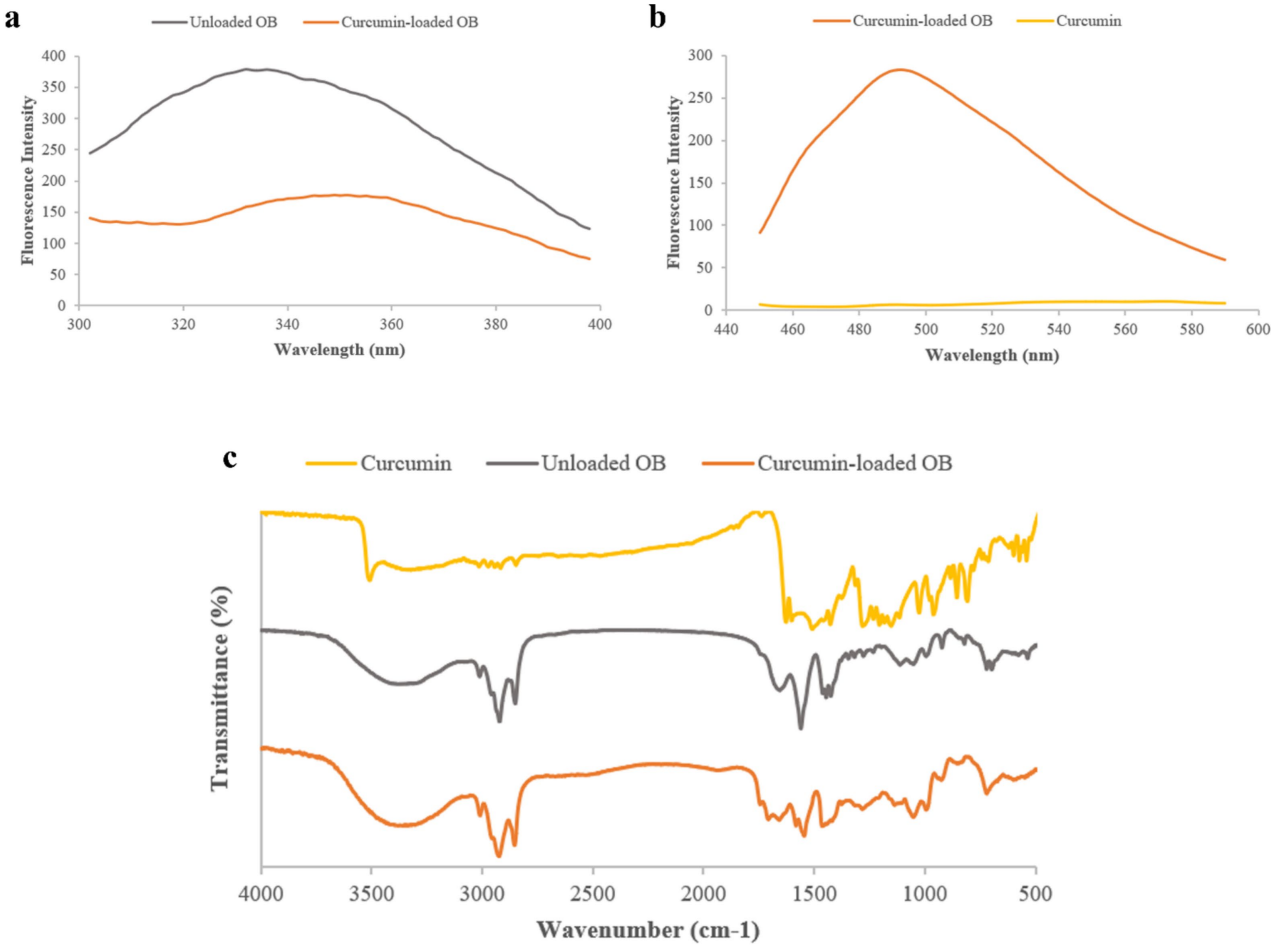
**(a)** Intrinsic fluorescence spectra of unloaded- and curcumin-loaded oleosomes (OB). **(b)** Fluorescence spectra of curcumin. **(c)** FTIR spectra of unloaded- and curcumin-loaded oleosomes.

Free curcumin exhibited maximum fluorescence at ~572 nm, which increased and blue-shifted to 492 nm upon encapsulation. This result confirmed the successful migration of curcumin into the hydrophobic oleosome core. Othman et al. also obtained the same results, and a blue shift was observed after incorporating curcumin into nanocapsules. This phenomenon was due to the polarity change in the medium of curcumin, suggesting the environment of curcumin is more non-polar (44). Fluorescence of oleosome proteins (280 nm excitation) showed decreased intensity and a red shift for curcumin-loaded oleosomes. A red shift in the λ _max_ showed the tertiary conformation change in the oleosome proteins, opened likely due to the pH-shift encapsulation process (10). As the protein unfolded, more hydrophobic patches were exposed to the environment, increasing the surface hydrophobicity. As Vardar et al. (22, 24) reported, a part of curcumin remains at the oleosome interface during encapsulation. Therefore, curcumin molecules, due to their interfacial activity, interacted with the interfacial proteins, decreasing the intensity of the λ _max_ of proteins of curcumin-loaded oleosome (11, 45) (see Figure 4).

### 3.4 Functional properties of unloaded and curcumin-loaded oleosomes

#### 3.4.1 Antioxidant activity

**Figure 5:**
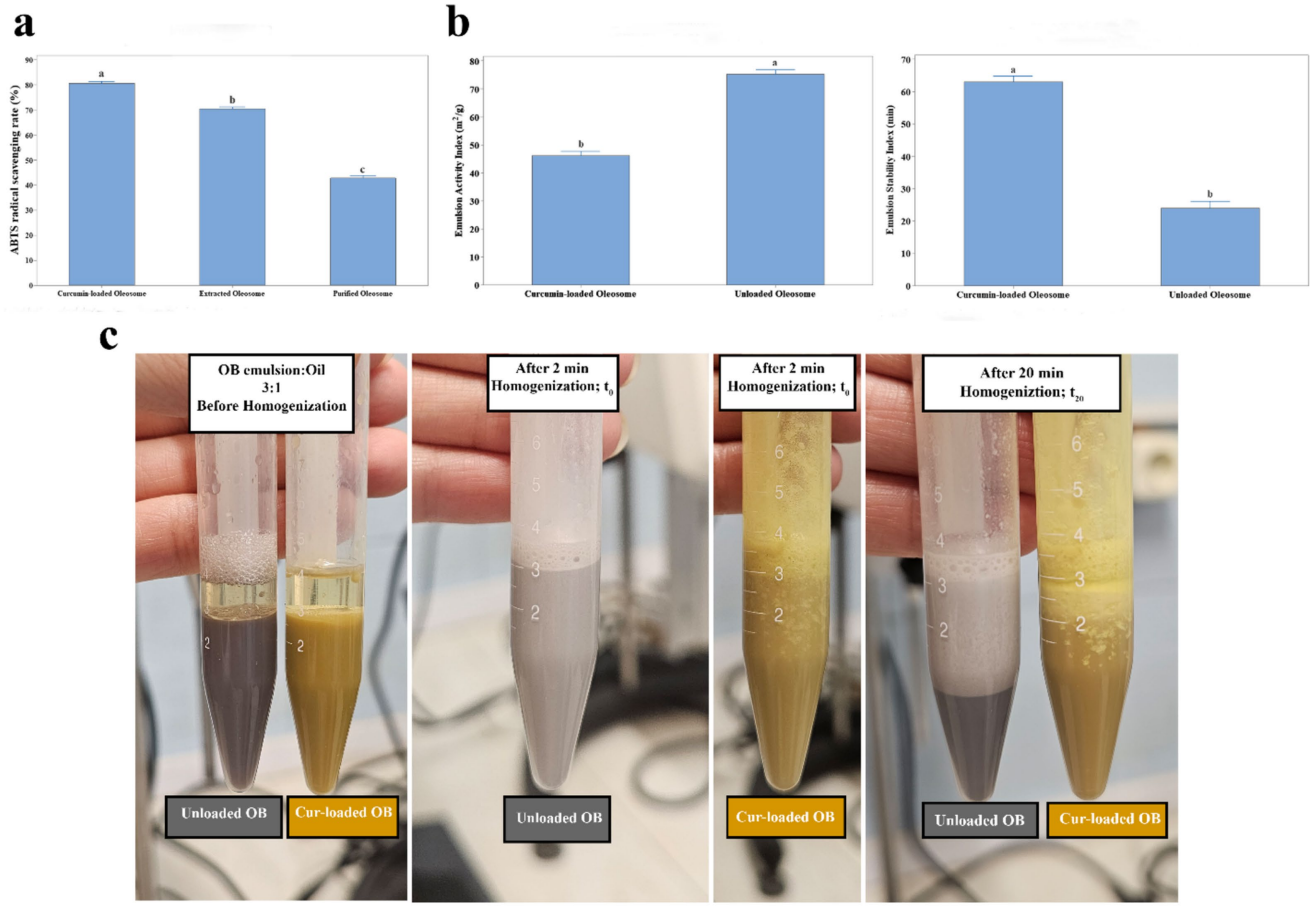
**(a)** Antioxidant capacity of extracted-, purified, and curcumin-loaded oleosomes (OBs). **(b)** Emulsifying properties of unloaded, and curcumin-loaded oleosome creams before and after 20 min (*p* < 0.05). **(c)** Appearance of unloaded, and curcumin-loaded oleosome creams before and after 20 min.

As shown in Figure 5a, purified oleosomes exhibited lower antioxidant activity (42.758% ± 1.692) compared to native oleosomes (70.341% ± 1.311), which may be due to loss of co-extracted intrinsic antioxidants, including not only storage proteins, but also phenolics, tocopherols, carotenoids, etc. According to Nikiforidis (6), native and unpurified oleosomes contain antioxidants that resist lipid oxidation, which makes native oleosomes have higher antioxidant activity than purified ones. In other words, the extraction methods could rarely result in completely pure oleosomes, which contribute to higher values of antioxidant activity of freshly extracted oleosomes (6, 46, 47). Incorporation of curcumin into oleosomes increased antioxidant capacity to 80.512% ± 1.418. This increase was attributed to the radical scavenging properties of curcumin, encapsulated well within black cumin oleosomes, maintaining the curcumin and its properties, which suggested the effectiveness of the pH-shift encapsulation method (48, 49). By the encapsulation process, it was found by Vardar et al. that curcumin locates not only within the triacylglycerol core, but it also partitions partly at the oleosome interface (11), which may contribute to the enhanced access of curcumin to the aqueous radicals.

#### 3.4.2 Emulsifying properties

The emulsion activity index (EAI) was 75.25 ± 2.82 m^2^/g for unloaded oleosomes and 46.26 ± 2.58 m^2^/g for curcumin-loaded oleosomes (*p* < 0.05) (Figure 5B). This decrease is attributed to changes in surface chemistry of oleosome droplets, consistent with the intrinsic fluorescence and the *ζ*-potential results. The reduced surface charge of curcumin-loaded oleosomes with more exposed hydrophobic patches to the environment caused them to associate with each other and thereby having reduced surface activity, which is caused by pH shift during encapsulation (50, 51). Another reason may be due to curcumin, covering the protein-phospholipid surfaces of oleosomes, resulting in a reduced effective interfacial area and emulsion activity (11). However, curcumin-loaded oleosomes displayed a higher emulsion stability index (ESI), ~39 min longer than native ones, likely due to exposure of more hydrophilic protein groups of oleosomes after the pH-shift encapsulation method, which was consistent with the intrinsic fluorescence results. The increased hydrophilic parts on the oleosomes, making them more hydrophilic emulsifiers, probably reduced interfacial tension between oil and water phases, and stabilized the oil droplets more within the o/w emulsions (52).

### 3.5 Curcumin release behavior

**Figure 6:**
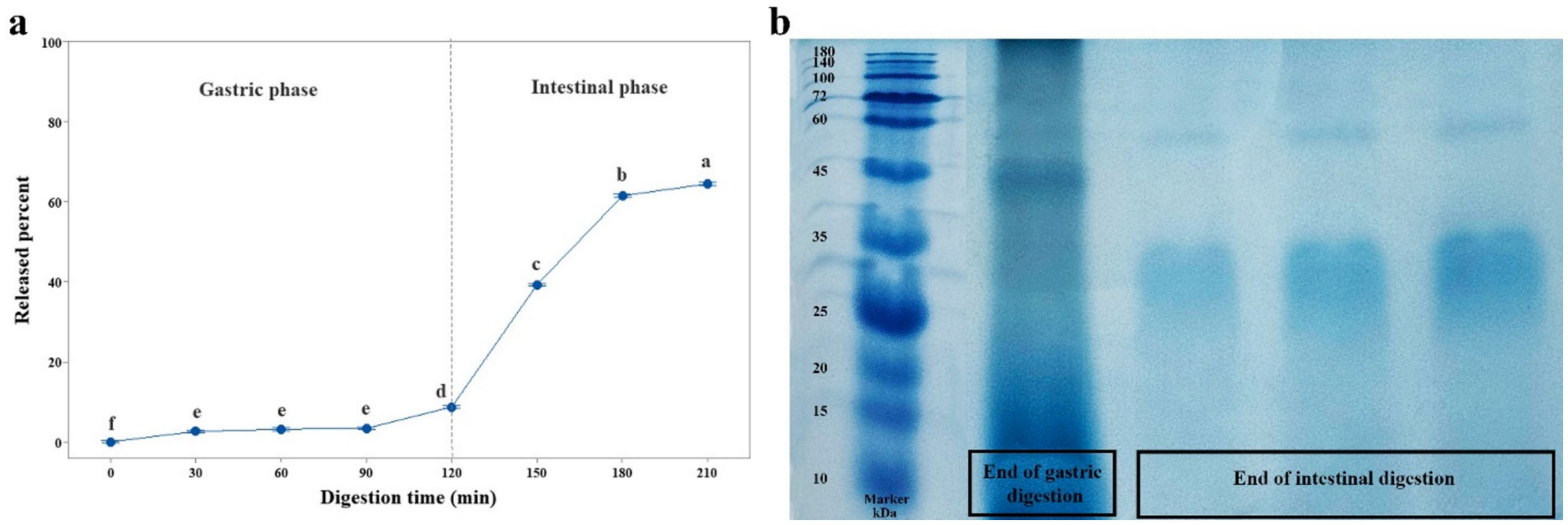
**(a)** Released percent of curcumin from curcumin-loaded oleosomes during simulated gastric and intestinal digestions. **(b)** SDS-PAGE profile at the end of gastric and intestinal digestion of curcumin-loaded oleosomes.

The release profile of curcumin from oleosomes under continuous simulated gastrointestinal digestion is shown in Figure 6a. During the gastric phase, curcumin was released slowly and progressively. The release ratio was initially only 2.63%, increasing modestly to 8.71% by the end of gastric digestion, which suggests the protective role of oleosome and its structural proteins, encapsulating the curcumin successfully. Under gastric conditions, oleosomes undergo structural changes, leading to instability of oleosome emulsions. Conditions like strongly low pH value, high ionic strength, and pepsin hydrolysis affect interface oleosome proteins, causing them to fold more, which decreases the exposure of charged groups and zeta potential values, weakening the electrostatic repulsion forces between droplets and causing them to aggregate (7, 8). The aggregated droplets are less exposed to the digestive environment, which is probably one of the reasons for the slow release of curcumin from curcumin-loaded oleosomes. Another reason may be due to the protection of the oleosome and its structural proteins. This protective effect is likely attributable to steroleosins, membrane-associated proteins that remained after gastric digestion and helped maintain oleosome integrity. Despite exposure to gastric digestion, steroleosins persisted and were only minimally hydrolyzed by pepsin (Figure 6b), thereby limiting oleosome decomposition. Consequently, the majority of curcumin remained encapsulated during the gastric phase.

Conversely, upon transition to the intestinal phase, a marked acceleration in curcumin release was observed, reaching 39.28% within the first 30 min. This increase is likely due to the penetration of bile salts and pancreatin into the oleosome membrane, which destabilized the oil droplets and promoted leakage of encapsulated curcumin (53). Another reason may be the intestinal environment, containing different anionic species such as bile salts and free fatty acids. Previously, Yimer et al. (19) and Kooti et al. (20) reported that the *ζ*-potential value of oleosomes becomes negative, as affected by anionic compounds. As a result, the repulsive forces between droplets increase, which results in a greater dispersion of droplets and a more exposed surface of them to the digestive enzymes. By the end of intestinal digestion, steroleosins were completely hydrolyzed, leading to substantial disruption of the oleosome membrane. Loss of structural integrity resulted in a final cumulative curcumin release of 64.47%.

Overall, these findings highlight the critical role of oleosome membrane composition in protecting encapsulated bioactives and enhancing the gastrointestinal delivery of nutraceuticals.

## 4 Conclusion

In this work, the black cumin oleosome, as a carrier from a novel source, was extracted to design a delivery system for curcumin. The modified alkaline extraction of oleosome successfully yielded ~38% with protein profiles preserved after washing and purification. Using the pH-shift method, curcumin was encapsulated within hydrophobic core of oleosomes with a maximum entrapment efficiency of ~ 90% at a 1:100 curcumin-to-oleosome ratio. Native (unloaded) and curcumin-loaded oleosomes were characterized. Curcumin-loaded oleosomes displayed wider and bimodal particle size distribution. The absolute value of *ζ*-potential decreased to 35 mV for curcumin-loaded oleosomes after pH-shift process. The results of intrinsic fluorescence confirmed the successful entrapment of curcumin within oleosomes and also revealed the conformational changes in oleosome proteins after encapsulation with curcumin being partly localized on the oleosome membrane. Additionally, curcumin-loaded oleosomes exhibited enhanced functional properties, such as antioxidant capacity improved to ~ 80%, and emulsion stability index achieved to 39 min longer stability. Moreover, the curcumin release during simulated gastrointestinal digestion was investigated, showing controlled release with released percent of ~ 9% and ~ 64% at the end of gastric and intestinal phases, respectively. Thus, these results demonstrated the efficacy of the pH-shift method for encapsulating curcumin in plant-derived oleosomes, maintaining its biological activities. However, this study investigated the incorporation of curcumin into oleosomes, and other hydrophobic bioactives should be selected to study further their encapsulation. Moreover, the releasing behavior of curcumin should be evaluated *in vivo*, using animal models.

## Data Availability

The original contributions presented in the study are included in the article/supplementary material, further inquiries can be directed to the corresponding authors.
